# t^4^ report*: Toward Good Read-Across Practice (GRAP) Guidance

**DOI:** 10.14573/altex.1601251

**Published:** 2016-02-11

**Authors:** Nicholas Ball, Mark T. D. Cronin, Jie Shen, Karen Blackburn, Ewan D. Booth, Mounir Bouhifd, Elizabeth Donley, Laura Egnash, Charles Hastings, Daland R. Juberg, Andre Kleensang, Nicole Kleinstreuer, E. Dinant Kroese, Adam C. Lee, Thomas Luechtefeld, Alexandra Maertens, Sue Marty, Jorge M. Naciff, Jessica Palmer, David Pamies, Mike Penman, Andrea-Nicole Richarz, Daniel P. Russo, Sharon B. Stuard, Grace Patlewicz, Bennard van Ravenzwaay, Shengde Wu, Hao Zhu, Thomas Hartung

**Affiliations:** 1 The Dow Chemical Company, Midland, MI, USA and Dow AgroSciences, Indianapolis, IN, USA; 2 School of Pharmacy and Biomolecular Sciences, Liverpool John Moores University, Liverpool, UK; 3 Research Institute for Fragrance Materials, Inc., Woodcliff Lake, NJ, USA; 4 The Procter and Gamble Co., Cincinnati, OH, USA; 5 Syngenta Ltd, Jealott's Hill International Research Centre, Bracknell, Berkshire, UK; 6 Johns Hopkins Bloomberg School of Public Health, Center for Alternatives to Animal Testing (CAAT), Baltimore, MD, USA; 7 Stemina Biomarker Discovery Inc., Madison, WI, USA; 8 BASF SE, Ludwigshafen am Rhein, Germany and Research Triangle Park, NC, USA; 9 National Toxicology Program Interagency Center for the Evaluation of Alternative Toxicological Methods, National Institute of Environmental Health Sciences, Research Triangle Park, NC, USA; 10 Risk Analysis for Products in Development, TNO Zeist, Zeist, The Netherlands; 11 DuPont Haskell Global Centers for Health and Environmental Sciences, Newark, NJ, USA; 12 Penman Consulting, Brussels, Belgium; 13 Department of Chemistry and Center for Computational and Integrative Biology, Rutgers University, Camden, NJ, USA; 14 US EPA/ORD, National Center for Computational Toxicology, Research Triangle Park, NC, USA; 15 University of Konstanz, CAAT-Europe, Konstanz, Germany

**Keywords:** computational toxicology, chemical similarity, read-across, hazard assessment, uncertainty

## Abstract

Grouping of substances and utilizing read-across of data within those groups represents an important data gap filling technique for chemical safety assessments. Categories/analogue groups are typically developed based on structural similarity and, increasingly often, also on mechanistic (biological) similarity. While read-across can play a key role in complying with legislation such as the European REACH regulation, the lack of consensus regarding the extent and type of evidence necessary to support it often hampers its successful application and acceptance by regulatory authorities. Despite a potentially broad user community, expertise is still concentrated across a handful of organizations and individuals. In order to facilitate the effective use of read-across, this document presents the state of the art, summarizes insights learned from reviewing ECHA published decisions regarding the relative successes/pitfalls surrounding read-across under REACH, and compiles the relevant activities and guidance documents. Special emphasis is given to the available existing tools and approaches, an analysis of ECHA's published final decisions associated with all levels of compliance checks and testing proposals, the consideration and expression of uncertainty, the use of biological support data, and the impact of the ECHA Read-Across Assessment Framework (RAAF) published in 2015.

## 1 Introduction

Over the last decade, the world has witnessed the introduction of new regulations on chemicals in several geographic regions and countries (for example EU, China, Taiwan, Korea, Turkey) that require companies to meet safety data requirements for their already marketed chemicals, often resulting in the generation of new toxicological data and the execution of a risk assessment to address any hazards identified. Within these regulations, the data needs are driven by some form of proxy for potential exposure (e.g., manufacturing or import volume) and as a consequence of the hazard and risk assessments undertaken, chemicals may be subject to restrictions on how they are used or, alternatively, phased out.

With the need to generate data to comply with regulations, read-across has the potential to play an important role in the hazard assessment of chemicals under numerous regulatory programs and consequently offers the potential for significant savings in terms of animal testing, product development time and costs. As an example, the Research Institute for Fragrance Materials (RIFM) is currently re-assessing fragrance ingredients based on the latest criteria document (Api et al., 2015). Read-across is one of the major data gap filling approaches that can be applied here. Of the 24 published fragrance ingredients' safety assessments, 20 of them (over 80%) used read-across to address/waive at least one endpoint (RIFM, 2015). Although there are many avenues where read-across can be utilized, acceptance of read-across by many regulators has been slow and unpredictable.

Over the past five years the chemical and cosmetic industries have worked to identify and attempt to address the challenges that read-across acceptance presents. Several projects and working groups have been established to identify opportunities to make read-across more robust, less uncertain and more available to a broader array of stakeholders (Berggren et al., 2015; Patlewicz et al., 2013a,b, 2014a,b, 2015). Several technical guidance documents (OECD, 2014; ECHA, 2008; ECETOC, 2012) aim to inform on the key considerations for preparing a read-across justification for regulatory purposes. This technical guidance has evolved and now offers many more practical insights about how one may approach and document a read-across justification, however, there still remains a lack of consensus about the extent and type of evidence needed to substantiate a read-across for a given endpoint.

Upon the request of its chemical / consumer product sponsor companies, the Johns Hopkins Center for Alternatives to Animal Testing (CAAT) started an initiative to facilitate read-across use. A white paper was developed to clarify the terminology and highlight the current short-comings associated with the use of read-across and potential opportunities for improvement (Patlewicz et al., 2014). The group recruited further expertise and developed, over the course of 2015, a compilation of the current state of the art as is presented here. Special focus was given to four aspects, i.e., to describe the breadth of tools and data available, to offer guidance on how to express uncertainty, to present proposals on how to use biological activity support data and to provide a compilation of feedback received from ECHA for read-across justifications that had been submitted in earlier REACH registrations. Two stakeholder fora, one in Brussels on February 26, 2016 (jointly with CEFIC and other stakeholders) and a similar one in Washington on March 1, 2016 (hosted by FDA), presented and discussed the derived insights.

## 2 State of the art of read-across

There is no single answer to the question of what the current state of the art of read-across is. To determine the success, or otherwise, of read-across as an alternative technique to animal testing for toxicological assessment, a non-exhaustive list of criteria was established to identify key points of reference. These are detailed below.

Read-across, at least conceptually, appears simple, i.e., the properties of two “similar” molecules will be similar. The known information on the property of a substance (source) is used to make a prediction of the same property for another substance (target) that is considered “similar”. In reality, the application of read-across is more difficult and often subjective. The crux of the matter, at least for regulatory use of read-across, is the burden of proof placed on providing evidence to demonstrate similarity for the property/endpoint for which a prediction is needed. The current state of the art is that a variety of well-defined scenarios are available to establish similarity, e.g., structural or mechanistic analogues, chemical similarity (including structural and chem- and bio-reactivity similarity), the formation of common metabolites, similarity of physicochemical property, etc.; these approaches are explained in several guidance documents (OECD, 2014; ECHA, 2008; ECETOC, 2012; Wu et al., 2010; Patlewicz et al., 2013a).

The confirmation that a chemical belongs to a group / category, or is similar to another chemical (analogue validity) is one of the key aspects to performing a read-across. Currently, it is widely acknowledged that evidence is required to demonstrate the validity of analogues, but the level of evidence required is not defined and even may be not (quantitatively) definable. As such, this is a stumbling block for regulatory acceptance. Current approaches to help confirm category membership are centered on the definition of uncertainty. There are many areas in a read-across where uncertainty can be identified and quantified in general terms (low, medium, high), the majority of which have been defined (e.g., Schultz et al., 2015; Patlewicz et al., 2015; Blackburn and Stuard, 2014), including the quality of the biological activity data, the read-across argument of similarity, etc. However, whilst levels of uncertainty could be defined, it is difficult to obtain regulatory acceptance of such approaches. This is because, in addition to defining uncertainty, there is also the question of how much uncertainty is acceptable for a particular purpose. Whilst it is understood that different levels of uncertainty would be required for, e.g., classification & labelling (C&L) versus hazard identification or risk assessment, the precise levels of uncertainty that may be deemed “acceptable” are not yet formally defined by regulators and are currently determined on a case-by-case basis.

A number of resources are available to support the use of read-across, and the state of the art of computational resources for determining chemical similarity is described further below. But, after chemical similarity has been established, the availability of high quality biological activity (e.g., toxicity) data is fundamental to the read-across prediction. Currently, a number of resources are available to read across from and support read-across, e.g., published literature, OECD QSAR Toolbox^1^, eChemPortal^2^ , the CEFIC AMBIT tool^3^, ECHA registered substances database^4^ as sources of *in vivo* data; ChEMBL^5^, Tox21^6^ ToxCast^7^ as sources of *in vitro* data, and various availabilities of toxicogenomics data. These resources, and many others like them, have greatly improved in accessibility and quantity of data over the past five years. However, there is limited advice on how to assess data quality with regard to read-across, and further improvements in chemical structure and data curation are urgently needed. Despite greater availability of data, there are still large gaps in the coverage of the chemical space, particularly with regard to high quality *in vivo* data – these cannot be addressed easily and consideration must be given to how to optimally use available resources. It should be noted that regulations like REACH (Regulation (EC) No. 1907/2006), which collect such high-quality *in vivo* and *in vitro* data, offer many opportunities to investigate read-across much further and promise to boost read-across opportunities (Luechtefeld et al., 2016a-d, this issue). In addition, as we enter the era of “big data”, i.e., datasets so large or complex that traditional data processing applications are inadequate, greater expertise and guidance is required on how to use these new datasets and the information they may contain in the appropriate context.

### 2.1 Specific use case scenarios

In addition to the formal grouping scenarios that may be attempted involving the generic categories noted above (e.g., analogue, common metabolite, etc.), there are also a number of use case scenarios that could, potentially, include all of the category formation methods. In consideration of the state of the art, it is important to take account of these as they impact on the overall success of read-across. For instance, read-across prediction is currently more accepted for predictions of the presence of toxicity, or for confirming membership to groups of specifically acting toxicants. Taking endpoints such as genotoxicity and skin sensitization as examples, the presence of a functional group for covalent reactivity (to DNA and proteins, respectively) could provide a clear and justifiable basis for grouping to perform subsequent read-across (Cronin, 2013; OECD, 2014; Blackburn et al., 2011; Wu et al., 2013). The situation is more difficult for receptor-mediated toxicities, and fewer grouping approaches based on receptor activation exist (Tsakovska et al., 2014). This is because the identification of toxicity primarily caused by the same receptor binding pathway for both the target and source chemicals is difficult with limited biological data. However, where such evidence for the presence of toxicity exists, it may provide a basis for read-across (Blackburn et al., 2015). More difficult at the current state of read-across is the development of groups and read-across for low or no toxicity: It is difficult to clearly identify the toxic/nontoxic boundary or activity cliff for a class of chemicals without detailed toxicity and potency data. Therefore, at this time, it remains difficult to confirm the absence of toxicity with low uncertainty, and indeed very few robust categories are available (Berggren et al., 2015).

Other more complex and possibly less resolved issues for read-across are associated with grouping for nanomaterials. A number of initiatives have attempted to rationalize grouping for nanomaterials (Arts et al., 2015; Bolt, 2014; Gebel et al., 2014; Oomen et al., 2015; RIVM, 2015; Walser and Studer, 2015), however, no overall consensus has been reached. Grouping (and any predictive modelling) of nanomaterials should not be confused with approaches for small molecules – many specific problems with data availability and quality as well as description of nanomaterials need to be resolved. Another very significant and currently very poorly addressed area of grouping is that of mixtures and substances of unknown or variable composition, complex reaction products or biological materials (UVCBs). Much more work is required in this area.

### 2.2 Applying read-across: Mechanistic basis and quantification

Read-across is most robust where it is transparent and allows a direct link to mechanism of action. The mechanistic links in category formation, starting from the chemical similarity, are crucial to support the foundation of read-across and aid in its interpretation. Currently, it is not always possible to assign a mechanism of action definitively. However, the last five years have seen growth in the uptake of Adverse Outcome Pathways (AOPs) and these now provide a possible mechanistic linkage. In addition, the AOP framework assists in the support of category formation by defining molecular initiating events as a measure of similarity (Mellor et al., 2016; Nelms et al., 2015) and raise the possibility of providing information on which (*in vitro*) assays, relating to key events, would be suitable to support read-across. The application of AOPs to support a mechanistic hypothesis in read-across is an attractive hypothesis but as yet largely unproven, and how to select/validate the large numbers of *in vitro* HTS data to elucidate the AOP is very difficult in reality.

Currently, read-across is best suited to the qualitative prediction of toxicity, as noted in the scenarios described in the previous section(s). Quantitative read-across is more challenging as more potential areas of uncertainty must be addressed. To make read-across more quantitative, as may be required for risk assessment, a number of issues must be addressed. The first is the realization that quantitative read-across may become akin to the use of quantitative structure-activity relationships (QSARs), which have been used to predict toxicity for many years (Cherkasov et al., 2014; Hartung and Hoffmann, 2009). Within small categories, trend analysis may also be a useful tool. Quantitative read-across is likely to work best where there is a definable and comparable endpoint, e.g., LD_50_, and it has been demonstrated for, e.g., acute aquatic toxicity (Koleva et al., 2008). There is also work on other, more challenging end-points, such as skin sensitization, to develop quantitative read-across approaches (Enoch et al., 2008; Roberts et al., 2008). The limitations to quantitative read-across include the availability of suitable quantitative data to read across from, lack of expertise on the use of alternative data to support the quantitative read-across as well as use of appropriate physico-chemical descriptors to base the quantification on (e.g., hydrophobicity for acute (eco-)toxicity and electrophilicity for skin sensitization, though both also depend on the reaction chemistry). Another little addressed issue is that of toxicokinetics (TK) to determine/assess the likelihood that the active chemicals or their metabolites (e.g., ultimate toxicants) can reach the target organ(s). Currently there is an understood need for better use of TK data (Ball et al., 2014) but little progress has been made. TK data will not only support the premise of category formation but may also be vital for quantification. Ensuring that there is sufficient fexibility to utilize and rely on *in vitro* TK data is also critical.

## 3 Regulatory acceptance of read-across

The acceptance of read-across varies between regions. For instance, read-across and analogous techniques are widely used as part of US EPA's Pre-Manufacture Notification Process (Cronin et al., 2003a,b). Within the EU, the acceptance of read-across for toxicity prediction in the regulatory context requires more understanding, and this topic forms the basis of much of the remainder of this paper. Notably, the EU REACH legislation explicitly calls for the use of non-animal alternative methods and thus opens up the use of read-across and ECHA's recently published Read-Across Assessment Framework (RAAF) is the first of its kind, strongly impacting on how read-across will be performed and evaluated in the future.

### 3.1 Analysis of REACH submissions

Approximately 75% of REACH dossiers contain read-across for at least one endpoint (ECHA, 2011, 2012, 2013, 2014). Considering that approximately 10,000 registrations were submitted over the course of the 2010 and 2013 registration campaigns, this indicates that a substantial number of hazard assessments are reliant on the integrity of the read-across approach and that registrants have been keen to take advantage of the potential reductions in vertebrate testing afforded. Notably, about 150,000 of the 850,000 study documents used for the REACH registrations were read-across / grouping approaches (Luechtefeld et al., 2016a, this issue).

Given the significant data requirements for the larger volume band registrations (> 100 t) it is no surprise that data requirements for endpoints such as reproductive toxicity, developmental toxicity and sub-chronic toxicity have most often been addressed with read-across or waiving approaches, with relatively few test proposals being made compared to the number of substances registered (Rovida et al., 2011; ECHA, 2011, 2012, 2013, 2014). The number of animals used to address these endpoints would be high (due to the production of litters within the reproductive and developmental toxicity studies) such that upwards of 5,000 animals would be required for each substance, not to mention the associated substantial financial and resource costs (Hartung and Rovida, 2009; Rovida and Hartung, 2009). Consequently, the successful application and acceptance of read-across is critical to meeting the goal of characterizing hazards of substances subject to REACH while minimizing new animal testing.

This then raises the question of how successful the application of read-across approaches in REACH has been. While the annual ECHA evaluation reports, fact sheets and practical guides provide general feedback on dossier quality and the use of approaches such as read-across, there are few documented examples of read-across successes and failures (e.g., ECHA, 2012). So, although it is often reported that significant challenges face the successful use of read-across under REACH (Patlewicz et al., 2014, 2015; ECETOC, 2012), it is difficult to understand what the common pitfalls are and what approaches have worked. With the recent publication of the RAAF by ECHA (2015), it is possible to view read-across from the assessor's perspective and subsequently infer what is needed to build a robust justification for read-across, and this will be discussed in more detail later. However, one thing that has become apparent with the publication of the RAAF is that only very low levels of uncertainty are currently accepted for a successful submission.

Until recently it has been difficult to locate actual case studies illustrating the practical implementation of read-across and whether they worked or not, but now ECHA has committed to publishing (with some redaction) the final decisions associated with all compliance checks and testing proposals. These have allowed a more detailed analysis of the success of read-across (and several other alternative approaches not discussed further here, e.g., exposure based waiving and use of QSARs) and the compilation of key findings and case studies to illustrate these.

The following analysis of read-across under REACH is based upon the compliance check (524) and testing proposal (388) final decisions that were publically available on the ECHA website^8^ as of July 31, 2015. The decisions were manually searched to identify those that included some reference to the use of read-across, either as proposed by a registrant, a third party or a member state during the course of the decision-making process. Based on the initial analysis, these decisions were then assigned to one or more categories based on the apparent cause for rejection of read-across. It is recognized that this analysis was not able to capture situations where a compliance check was initiated and subsequently terminated due to the registrant addressing the initial concerns. According to the ECHA evaluation reports (ECHA, 2011, 2012, 2013, 2014) 879 (out of 1,658) compliance checks were terminated prior to reaching a final decision. It is possible that some of these involved the use of read-across and that the information provided by the registrant was sufficient to address any concerns raised by ECHA, leading to the conclusion of compliance (at least with respect to the focus of the initial draft decision). There are also a substantial number of dossiers where a read-across approach has been used but no formal assessment by ECHA has taken place that would result in a final decision that would be disseminated. Consequently, the analysis of publicly available compliance checks and testing proposals only refects a small part of the overall picture on the use of read-across, but nevertheless is illustrative of the challenges and possibilities that registrants face.

Approximately one fifth (107) of all disseminated compliance check decisions involved the use of read-across. Of these only one or two appear to have been accepted. The reasons for the rejection of the use of read-across fall into four main categories. These are shown in Table 1 (note that some cases were rejected due to a combination of reasons).

With respect to testing proposals, 81 out of 388 testing proposals involved the use of read-across (either presented by the registrant or by a third party during consultation on the testing proposal). In proposals submitted by the registrant, a category (or analogue) testing plan was proposed where some members of the category would be tested and the data from these studies would then be used to read-across to the other category members. Test proposals appear to have been far more successful in the use of read-across, with 50 approved at least in some part. Out of this group, two proposals to test an analogue were accepted for some endpoints but not others: In one case, the *in vivo* mammalian testing (a 90-day study) using an analogue was accepted but an earthworm toxicity study was not, illustrating the potential differences in read-across justification between environmental and human health endpoints; in the other case, the developmental toxicity study using an analogue was accepted but the 90-day study was not, although the acceptance for the developmental toxicity was contingent on the outcome of a toxicokinetics assessment.

It should also be noted that many of the decisions still include a reminder to the registrant that the approval of the testing plan should not be interpreted as approval of the use of read-across. Rather it is up to the registrants to ensure that once the data are generated, they review the read-across strategy to determine if it is still appropriate. Consequently, once the approved studies are submitted, the use of read-across would still be reviewed as part of a dossier evaluation, leading to future compliance checks and potential later rejection of the read-across.

Of the testing proposals in which the use of read-across was proposed and subsequently rejected, approximately half involved proposals for the use of read-across made by a third party during the public commenting period on testing proposals. While some of these proposals may have had some merit, the lack of information provided in the third party proposals led to their rejection. This perhaps refects a lack of sufficient understanding and rigor on the part of some interested third parties combined with the lack of access to the toxicological databases for potential analogues.

In the remaining cases where the registrant proposed read-across and it was subsequently rejected, the reasons for rejection were consistent with those associated with compliance checks, namely lack of supporting information and scientific plausibility.

Based on the analysis of the compliance checks and testing proposals, i) the lack of sufficient supporting information, ii) the scientific plausibility, and iii) challenges relating to substance identity represent the areas where additional clarity could help with increasing the future quality of read-across justifications. Each is addressed in turn with some case studies taken from the pool of compliance checks and testing proposals to illustrate specific issues that commonly occur or appear to present the most significant barriers (Tab. 2).

#### i) Lack of sufficient supporting information

In several instances, registrants had referred to data on a source substance to address the data gaps as part of a read-across approach. However, the data on that source substance was not included in the submission (Case 1: 2-diethylaminoethanol; Case 2: reaction mass of amides, rape-oil, N-(hydroxyethyl), ethoxylated and glycerol, ethoxylated). In principle, addressing this issue appears straightforward. Ensuring all necessary data are included in the dossier with the appropriate explanation of what each study is providing in terms of support for the use of read-across is a somewhat simple but critical element to the preparation of a read-across justification. References to the data on another substance that are not provided are almost never accepted as support for read-across. However, securing access to the data supporting a read-across case has implications for purchasing access to data and where data are not publically available the cost of purchasing studies on multiple substances to support the use of read-across may be prohibitive to registrants.

In other cases, unsubstantiated statements that form the basis of the hypothesis have led to rejection of read-across. For example the hypothesis is substance A (the target) is rapidly metabolized to substance B (source), so data on substance B can be read across to substance A. However, no metabolism data are provided and thus the statement regarding the metabolic pathway and its rate is unsubstantiated (Case 3: dipropylene glycol methyl ether acetate). In this case study, *in vitro* metabolism data was subsequently provided during the compliance check, but was considered insufficient to support the use of read-across (ECHA, 2012).

In another case (Case 4; cycloexyldimethoxymethylsilane), the use of read-across was rejected because the justification did not take other metabolites generated into consideration and the registrant did not provide sufficient information to substantiate the statement about the primary toxicant. In this case it was hypothesized that the analogues would release methanol and that this would be the primary toxicant. While this was considered to be likely (that all produce methanol) there were no data provided to support the assertion that only methanol would drive the toxicity. Unfortunately, the information necessary to substantiate this statement could be extensive and involve incorporating the full datasets of the primary metabolite (e.g., methanol) so that available data on the substances of interest can be compared to it, as well as some form of assessment about the toxicity of other potential metabolites. While this may be possible, it becomes clear how challenging it can be to substantiate a simple statement such as “This metabolite will drive the toxicity.”

#### ii) Scientific plausibility

Where the scientific plausibility of the use of read-across was challenged, the root cause was very case-specific and often resulted from a combination of insufficient information to support the read-across hypothesis and inadequate/conficting/inappropriate information provided by the registrant. Metabolism featured in several case studies as a contributor to the scientific plausibility assessment. For example, in one case (Case 5: ethylene carbonate) the metabolism was considered to be too slow to support the hypothesis for the use of read-across. In another (Case 6: 4-hydroxy-4-methylpentan-2-one), concerns about potential differences in the rate and extent of metabolism between routes (inhalation versus oral) led to uncertainty about the relevance of data generated with an analogue via one route to the target substance. This latter case highlights that when preparing a read-across justification that relies on data generated via different routes of exposure, toxicokinetic data via all relevant routes will contribute to and may be required in the assessment. It should be noted that this TK data could either reduce the uncertainty in the read-across or it could give rise to significant uncertainty. Regardless, given the paucity of experimental toxicokinetic data on industrial chemicals, the requirement for data generated via multiple routes could present a significant challenge to the application and acceptability of read-across as an alternative method for some chemicals.

In another case (Case 7: 2-butene), metabolism was not sufficiently addressed as a potential contributor to divergent toxicity. In particular, the assessors felt that the different placement of a double bond within the category members could lead to significant differences in toxicity and this had not been addressed by the registrants. It is important to note that the chemical, bio-reactivity and mode of action of cumulated double bond, conjugated double bond and the position of the single double bond in the chemicals are not similar. Use of a less active chemical to read across a more active chemical would certainly increase uncertainty. Where data were provided to support similarity in the toxicity profiles there was one case where the data were considered to be inadequate/not relevant or appeared to undermine the grouping approach (Case 7: dibutyl fumarate). The registrant had referred to data on several other endpoints as supporting data for the read-across justification but the differences in toxicity observed in these other endpoints led to the concern that the toxicity of the group was not consistent, consequently the validity of the read-across was questioned. In a final example, the use of read-across involved extrapolation of data rather than interpolation and this was considered to give rise to too much uncertainty (Case 8: hydrogenated dimerization products of 1-decene, 1-dodecene and 1-octene). These cases highlight the potential challenges of providing effective anchor data for the read-across; particularly where it concerns the need for data on specific endpoints when read-across is being utilized.

#### iii) Substance identity

The definition of substance identity for well-defined mono-constituent substances is usually straightforward and hence has not been a significant reason for the rejection of the use of read-across in such cases. Conversely, where multi-constituent or UVCB substances are involved, substance identity is more complex and has been a significant roadblock to the acceptance of a read-across approach. As specified in Annex XI in the REACH legal text^9^:

“Substances whose physicochemical, toxicological and ecotoxicological properties are likely to be similar or follow a regular pattern as a result of **structural similarity**^10^ may be considered as a group or category of substances.”

Consequently the use of read-across begins with structural similarity. When assessing read-across between well-defined mono-constituent substances, the differences in structure between the target and source(s) is often a significant contributor to the uncertainty associated with the use of read-across. How do any differences in structure contribute to the toxicity profile? Could these differences lead to a divergence in toxicity? Addressing these questions can be challenging for a category of mono-constituent substances (Patlewicz et al., 2014). Consequently, where the composition of a substance is unknown or variable and the structures of the constituents are not well characterized, it is very difficult to demonstrate that two such substances are structurally similar and to address the questions about how differences in composition and differences in structure between constituents could impact the toxicity. Within the pool of compliance check decisions and test proposal decisions there are several that involve the use of a read-across strategy for categories of UVCB substances. In some cases the compliance check did not result in the rejection of the use of read-across, but rather a request for better substance identity information was made so that a subsequent assessment of the use of read-across could be performed (e.g., hydrocarbon solvents, petroleum substances, resins and rosins). With respect to the testing proposals, there are several examples of where the use of read-across was approved in principle along with the testing of specific category members. However, as indicated above, the requirement for registrants is to ensure that the case for read-across, including the provision of more robust substance identity information, is still relevant after the data are generated. In these cases it is still possible that further compliance checks are made once dossiers are updated.

If one considers the products of the oil and refining industry as a representative group of UVCB substances, one will find that there are over 400 individual CAS numbers for these substances and read-across has played an important role in their hazard identification. The majority of these substances are produced or marketed in a yearly volume of > 1,000 tons, each requiring all the higher tier animal (and potentially ecotoxicity) studies. If a way forward in addressing UVCB categories is not forthcoming, then the potential need for testing could be significant. Unfortunately there is no straightforward answer to the issue of characterizing the sameness of UVCB substances, and while there are analytical approaches (e.g., characterization of functional groups present^11^), it may also be possible to utilize biological profiling approaches to support the concepts of substance sameness. The concept of biological profiling is covered in some additional detail in a later section.

### 3.2 What worked?

As indicated above, there were cases where the use of read-across was accepted, particularly when it came to the testing proposals. The successful applications of read-across are pre-dominantly associated with category testing proposals with four categories making up the majority of the accepted testing proposals.

One of these involved metal compounds, where the toxicity of the compound was tied to the metal and the bioavailability of the metal (Case 10: cobalt compounds). The registrants provided sufficient data on the characteristics that impact bioavailability to support the hypothesis for which substances would be most and least toxic, and proposed to test the most and least bioavailable members of the group, allowing the use of interpolation for the remaining category members.

In a second category (Case 11: higher alpha olefins), test proposals for 19 substances were accepted for several higher tier human health studies. After an initial proposal for testing was rejected, a revised strategy was proposed, where several members of the category would be tested. The overall hypothesis for the use of read-across included bioavailability as a driver for toxicity and also a general assessment of low overall systemic toxicity. It was argued that as bioavailability decreases, systemic toxicity also decreases and that this decrease in bio-availability can be associated with molecular weight. Testing was proposed on substances both at the extremes and within the category based on bioavailability. To support this argument, *in vitro* bioavailability data were generated on 33 members of the category using inverted rat gut sacs (Penman, 2015). The approach was accepted, but as with some other cases, the registrants were reminded that the updated dossiers should include a robust category justification that addresses all areas of uncertainty that were identified during this process.

## 4 What next?

With an understanding of the state of the art of read-across and some important experience gained through the REACH Regulation, we find ourselves in a position where there is some understanding of the available tools and where we appear to be failing/succeeding. The question therefore remains, how can the tools be better applied to increase the quality of read-across justifications (not just for EU REACH but also beyond)?

### 4.1 Substance identity

Substance identity will continue to be a significant barrier to the acceptance of read-across within groups of UVCB substances. There have been some notable successes for these categories, including the hydrocarbon solvents and the resins and rosins, where test proposals have been approved for certain category members. However, these substances likely will still face further scrutiny once the data are generated such that future compliance checks may follow. At this time it is difficult to see a way around this particular impasse where ill-defined UVCB substances must be grouped based on structural similarity for the purpose of read-across, but where the level of detail about composition is difficult to achieve in a way that satisfies the expectations of the “structural similarity” assessment. The creation of a biological profile for these types of substances using conventional data and *in vitro* data from high-throughput/ high-content assays may provide a means to support the overall concept of similarity, but it would require an acceptance of the uncertainty associated with the lack of a precise compositional comparison between members.

### 4.2 Providing sufficient supporting information

As indicated earlier, ensuring that the appropriate data on analogues/category members is included in a dossier (in the form required by the regulator) is critical. Having accomplished this, the task remains to identify what other data could be utilized to support the read-across justification and to ensure that the information is reliable, relevant and presented in such a way that it is clear how it supports the overall read-across justification.

#### 4.2.1 Potential sources of supporting information

Patlewicz et al. (2015) present an assessment of how one can build confidence in the use of read-across; presenting several considerations that should be taken when preparing the justification, with guidance on the types of supporting information that can be included, specifically, cheminformatics and biological data. Although there is substantial guidance on which cheminformatics tools are available and how to utilize them effectively (ECETOC, 2012; ECHA, 2008; OECD, 2014), it is still important to understand their potential limitations and the expectations of how the information should be reported. With respect to novel biological data, there is still relatively little consensus on how this is best used in support of read-across. These two areas are discussed separately below with considerations for ensuring that data meet the quality, reliability and relevance expectations.

#### 4.2.2 Cheminformatics

In general, one can consider that the primary functions of cheminformatics tools in the field of read-across are analogue identification, information retrieval, and the initial prediction of some properties (including physicochemical ones and toxicokinetic parameters such as bioavailability and metabolic pathways). In essence, these tools can provide the basis for why substances should be considered to be in a category and potentially identify those that should not be included. It is noteworthy that these tools are of limited use for UVCB substances unless one can identify sentinel compounds within the composition that could act as a proxy for the rest of the substances.

When considering the use of cheminformatics information (e.g., QSAR) there are very clear expectations from regulators (e.g., ECHA guidance on use of QSAR^12^) as to how the information is reported, whether the substances for which predictions have been made fall within the applicability domain for the tools used, and whether the tools are actually considered to be capable of making a reliable prediction for a given endpoint. Therefore, when making use of these tools, ensuring one can meet these standards is a pre-requisite.

There are many pieces of software and tools to assist the practitioner in various aspects of grouping and read-across, from assessing chemical similarity and retrieving biological data to creating a report. Good reviews are available on computational tools with a focus on read-across, e.g., from JRC^13^, OECD^14^ and numerous review papers cited in this guidance as well as the output from the EU Project Antares^15,16^ . One area where more attention is particularly needed is metabolism prediction, as the process is inexact and does not lead to quantifiable prediction of relevant metabolites (Kirchmair et al., 2015).

At the moment, the most commonly utilized software is the OECD QSAR Toolbox^17^. At the time of writing, this tool is undergoing considerable revision and version 4 of the Toolbox is due for public release in 2016. Perhaps one area in the realm of read-across and cheminformatics that would benefit from some attention is actual worked case studies of how to use tools such as the OECD toolbox to facilitate the creation and support the use of read-across. For instance, there are several examples of how the OECD toolbox can be used to predict endpoints such as genotoxicity and skin sensitizing potential (e.g., Patlewicz et al., 2015) using a hybrid read-across/QSAR approach, but with respect to using them to create a category for multiple endpoints, there are still very few (if any) examples of how this should be done. Potentially the case studies coming from the SEURAT project (Berggren et al., 2015) may provide this, but if not, more case studies would be of great benefit to practitioners of read-across.

With respect to the quality/reliability/relevance of cheminformatics information, one consideration that is rarely raised or addressed is that pertaining to the quality and reliability of the information that forms the basis of the QSAR models. Any cheminformatics study involves producing chemical descriptors that are expected to accurately refect underlying chemical structural details. Errors in the structures would inherently translate into either an inability to calculate descriptors for incorrect chemical records or into erroneous descriptors, ultimately resulting in either restrictive or potentially inaccurate models. There have been a number of studies that have analyzed the error rate in structural databases (Williams and Ekins, 2011; Williams et al, 2012; Karapetyan et al, 2015) and the subsequent consequences on predictive power from both random and systematic errors, and found, unsurprisingly, that careful expert manual curation significantly increases QSAR model predictive power (Tropsha, 2010). As a user of a QSAR tool, it will be difficult to assess whether the tools are built using reliable, well-curated data. As such, in order to maintain the utility of QSAR tools either for predictions in their own right or to support a grouping/read-across approach, it is critical that the quality of the information going into these tools is closely monitored, particularly as a wider diversity of data (for example ToxCast data) are used to build models.

#### 4.2.3 Biological data

The wide range of tools that fit within the concept of biological profiling/biological activity assessment has the potential to be used not only to provide insight into potential toxicity, complementing existing *in vivo* data within a category, but also as a means to support and defend the category composition. Using these types of tools to demonstrate that a group of structurally similar substances can be grouped together based on both their structural and biological similarity could be a powerful tool to support the use of read-across. An overview of what could be considered the “state of the art” for biological tools supporting read-across is presented in a parallel paper (Zhu et al., 2016, this issue), but a short overview is provided herein for completeness and to illustrate the utility of these tools with respect to read-across.

As indicated above, chemical similarity, including structure, reactivity and physicochemical property similarities, is the main approach so far employed to justify read-across. Toxicity, however, is primarily a biological response, so the biological similarity could also be used as a basis for similarity and thus to justify read-across. Three different types of approaches are available: (1) Biological assays can represent key events directly and thus have predictive value; in some instances it has been possible to show this for a larger applicability domain by traditional validation; some methods, however, work well for certain parts of the chemical universe, which has been termed “local validity” (Patlewicz et al., 2014). (2) Large curated datasets allow characterizing biological similarity based on a multitude of biological assays. This avenue is so far rarely pursued, but the more recent availability of such larger datasets of curated and more or less standardized biological assays such as the ones of ToxCast or Tox21 open up such opportunities (Kleinstreuer et al., 2014). (3) Complex biological systems, which represent many biological, possibly perturbed pathways, combined with holistic omics analysis also permit an assessment of similarity; here *in vivo* models, such as short-term animal studies as well as stem cell-derived developmental and organ models, lend themselves for signatures of toxicity to be compared (Zhu et al., 2016, this issue).

#### 4.2.4 Use of *in vitro* assays of local validity

Toxicological properties are not randomly distributed in the chemical universe. Certain functional groups (structural alerts, in the case of an effect), chemico-physical properties or simply molecular sizes make a property likely or unlikely. Other areas may show activity cliffs, i.e., sudden changes in properties with small changes in structure. For this reason, areas of relative certainty for read-across, *in silico* methods (Cronin et al., 2003b) or, following the same concept, for *in vitro* assays can be defined. This means that very reliable predictions can be obtained for a certain applicability domain (Hartung et al., 2004). The term “local validity” was introduced to describe this concept (Patlewicz et al., 2014). In consequence, it will be possible in these areas of local validity to augment read-across by carrying out select *in vitro* tests that represent key aspects of the pathophysiology, a concept earlier introduced as test-across (Hartung, 2007). For example, substantial efforts have already been undertaken to develop alternative assays for the assessment of reproductive/developmental toxicity (Adler et al., 2011; Leist et al., 2014). Given the substantial amount of testing required in this area under the REACH regulation, this endpoint represents the largest opportunity for a win in terms of reducing the need for new animal studies. By combining an *in vitro* battery of tests with existing *in vivo* data on the members of a category there would be the opportunity to demonstrate consistency in terms of activity (presence or absence) for this endpoint. However, the complexity of the validation of any such battery of tests should not be underestimated, particularly where the prediction of absence of toxicity is desired.

#### 4.2.5 Using “big data” to establish biological similarity by comparative profiling of chemicals

The term “big data” is used for large datasets that only can be exploited by computer-assisted methods. One source of big data comes from high-throughput screening of large libraries of compounds in biological assays. There has been a huge increase in the number of compounds and associated testing data from different *in vitro* screens. Besides that, there are also efforts to curate historical *in vivo* toxicity data to share with the public. Examples of available sources of biological data are given in the parallel paper on biological support to read-across (Zhu et al., 2016, this issue).

Information regarding the biological properties of chemicals, both target and analogue, could be a key support piece to read-across. One approach is to use the results from a large number of assays, usually high-throughput assays, to profile the bio-fingerprint of a chemical. If two chemicals have similar bio-fnger-prints, they will be considered to be biologically similar (Low et al., 2013; Zhang et al., 2014). However, it is worth noting that biological similarity should serve as a “weight of evidence” to enhance the read-across and structural similarity will still usually be the first tier for similarity criteria.

The advent of high-throughput screening and research initiatives such as Tox21 and ToxCast provide data on a range of targets and pathways that may be linked to toxicity. The ToxCast dataset^18^ in particular affords a unique opportunity to attempt bioactivity based read-across (BaBRA) owing to the wide coverage of biological space and range of assays from different cell types, species and technology platforms. A number of predictive models have identified critical pathways. Low et al. (2013) described an approach that is pathway-agnostic and more closely resembles traditional structure-based read-across, with the addition of all available *in vitro* assay data as features to determine biological similarity. ToxCast *in vitro* assay data was used (Zhu et al., 2016, this issue) to perform BaBRA to predict *in vivo* endpoint information for chemicals by using data from the same *in vivo* endpoint from another chemical, which had similar *in vitro* activity. This bioactivity-based similarity was also enriched with structural similarity (St.BaBRA) and used to make predictions for a chemical's *in vivo* toxicity based on its nearest neighbors. The BaBRA and St.BaBRA predictions were produced based on a variety of proximity matrices, and compared to a range of *in vivo* endpoints. BaBRA and St.BaBRA are approaches that show great promise within certain applicability domains and well-curated datasets.

A complementary approach termed GenRA has used bioactivity, chemical descriptors and a hybrid of the two to make *in vivo* predictions for a range of different repeated dose toxicity study types. Performance was context-dependent on the groupings derived and the study outcome being addressed – highlighting how important analogue identification and evaluation are in the read-across process (Shah et al., 2016; Patlewicz and Fitzpatrick, 2016). However, broad *in vitro* activity patterns across a wide range of assays are difficult to correlate with apical *in vivo* toxicity endpoints, even when enriched with structural similarities. This is also complicated by the lack of metabolism capability in many high-throughput assays (Zhu et al., 2016, this issue). Feature selection and optimization methods should be explored to improve predictive accuracy and applicability. For example, identifying features that provide the best separation between positive and negative space for each endpoint in combination with *in vivo* data curation will improve the applicability of read-across. Further, biological pathway knowledge can be used to define the assay/proximity space that is relevant to the endpoint of interest.

#### 4.2.6 Using omics approaches to establish similarity for read-across and grouping

Standardized techniques and a database with reference compounds for application of grouping with data-poor chemicals are prerequisites for using omics technologies for read-across. Toxicogenomics (TGx) aims to study the underlying molecular mechanisms of toxicity and address challenges that are difficult to overcome by conventional toxicological methods by integrating genomic technology with bioinformatics. Several publicly available sources of TGx data such as the Japanese Toxicogenomics Project (TGP) (Uehara et al., 2010), DrugMatrix (Ganter et al., 2005), the NIH LINCs project^19^ and PredTox (Suter et al., 2011) provide enormous opportunities to evaluate and investigate a large set of TGx assays from a systematic point of view, which gives a landscape of TGx and more objective understanding of mechanistic of toxicity. Incorporating TGx into *in vivo* and *in vitro* studies and comparing the TGx profiles between a set of similar materials and (potentially) against publically available databases may provide the opportunity to demonstrate biological similarity or predict potential toxicity. This is essentially the same approach that has been investigated by BASF and Metanomics but using a similar technology, metabolomics. They have established a standardized metabolomics technology and built up such a database (Meta-Map^®^ Tox) with about 600 compounds administered to rats in repeated dose studies (van Ravenzwaay et al., 2014). The toxicological activity of data-poor chemicals in rats can be assessed by a standardized evaluation procedure with this database based on profile strength, pattern ranking, treatment correlation and pathway analysis. As a result of the mentioned evaluation process, an assessment can be made regarding 1) target organ toxicity, 2) systemic toxicity mode of actions by comparison with reference compounds, and 3) which pathways or which chemical groups of metabolites in the rat physiology are affected. The assessment is restricted to the set of reference compounds in the database and the established metabolite patterns defining which mode of actions can be covered.

With respect to the quality and relevance of biological data used/generated to support the use of read-across, there are several points to consider in addition to what is mentioned above. With many of the potential biological profiling techniques still being somewhat experimental, a clear challenge to using them in the future is demonstrating their reliability, reproducibility and relevance since biological data is often fraught with uncertainty and issues with reproducibility. An example of this comes from a recently published database of rodent uterotrophic studies, a screening test for estrogenic activity based on uterine cell proliferation, where 670 articles were analyzed in an attempt to identify high-quality data (Kleinstreuer et al., 2015). The extent to which the study protocols could be considered “guidelinelike” was assessed by multiple reviewers based on adherence to a set of minimum criteria from internationally harmonized test guidelines from US EPA and the Organization for Economic Cooperation and Development (OECD). Approximately 18% of the studies met all of the minimum criteria to be considered guideline-like studies; however, for those chemicals with multiple guideline-like studies, 26% of cases had both positive and negative uterotrophic results that were attributable to study design elements, such as dosing, in some cases and in other cases seem to be a reflection of the inherent variability in the animal data, even after study quality assessment. However, if study quality, reproducibility and biological relevance are all taken into consideration, one can achieve a high degree of accuracy in predicting toxicity endpoints from structural and biological similarity. It should be noted that study data used to support a read-across should be reviewed against the most recent guidelines on the generation of such data, i.e., protocol design, and interpretation of study outcomes such as these are subject to periodic review and a study call may change as the influence of confounding factors, e.g., use of high test substance concentrations or cytotoxicity, is considered versus the most up to date scientific consensus. Noteworthy, a particular study outcome can vary depending on the time when a study was conducted, i.e., the revision of test guidelines and methods (e.g., the revision of the top concentration/dose requirements in genotoxicity studies), the tightening up of cytotoxicity measures or introduction of the global evaluation factor in *in vitro* mammalian gene mutation assays and improvement of historical control data (HCD) compilation and use may all lead to older “positive” studies now being considered as negative. This may lead to a difficult situation where a registrant gathers data previously formally considered positive, but a modern interpretation is actually for a negative result. Hence, some older positive studies may perversely now support a negative read-across position; this is a challenge to communicate.

Another important aspect is the quality of reporting *in vivo* data as was elaborated in the ARRIVE guidelines^20^ (Kilkenny et al., 2010). Similarly, the Good Cell Culture Practice guidance, GCCP, (Coecke et al., 2005) established advice as to both the quality assurance of cell culture work and its reporting. Noteworthy, GCCP has been recently revived by CAAT by creating the International GCCP Collaboration with the aim to update the guidance and expand it to stem cell-based models and micro-physiological (organ-on-chip) systems; two workshops in 2015 and longer-term ongoing work by 90 experts on *in vitro* reporting standards are the first elements toward GCCP 2.0.

A number of public initiatives to address the issues of data quality, relevance, risk of bias, and reproducibility are ongoing, including the Evidence-based Toxicology Collaboration (EBTC)^21^ (Hoffmann and Hartung, 2006; Hoffmann et al., 2014) and the NIH Initiative to Enhance Reproducibility and Transparency of Research Findings and the associated formal approach to systematic review developed by the National Toxicology Program (NTP) Office of Health Assessment and Translation (OHAT)^22^. OHAT strives to apply transparent, rigorous, objective and reproducible methodology in literature-based evaluations to identify, select, assess and synthesize results of relevant studies (Rooney et al., 2014; Thayer et al., 2014). EBTC has developed the “ToxRTool” to assign Klimisch scores (Schneider et al., 2009), a measure of study quality and reliability (Patlewicz et al., 2015). These types of methods provide much-needed transparency for understanding the critical studies and increasing the overall confidence in a weight of evidence submission (Linkov et al., 2015). Models that rely upon chemical and biological data to make predictions may only be as good as the data itself. Therefore, there is a distinct need to further develop and employ chemical record curation, structure standardization, robustness analyses and systematic review protocols that will assist in processing chemical and biological datasets to increase confidence in model building and read-across approaches.

### 4.3 Scientific plausibility

Once substance identity has been addressed and every effort has been made to include the relevant supporting and key data on the different analogues/category members, scientifically supporting every statement made in the use of read-across justification becomes the most challenging barrier to the acceptance of read-across. This is mainly due to the fact that there are typically gaps in the information needed to comprehensively support read-across, whether it is substantiating statements relating to metabolism, the mode of action, or the aspect of the substance that drives the toxicity. Consequently the existence of uncertainty goes hand in hand with the use of read-across and there is a very real need to find a meaningful way to work with uncertainty in the assessment of read-across.

#### Uncertainty

The regulatory guidance available on read-across to date has been broad and has not resulted in a consensus on what constitutes a universally acceptable best practice for read-across that maximizes robustness and minimizes uncertainty associated with the use of data on one chemical to assess another. Therefore, continued efforts are needed to define the type and realistic extent of supporting evidence required to increase the scientific robustness of read-across and to improve transparency in the documentation of read-across. Both of these efforts will ultimately reduce uncertainty and improve the acceptability of read-across assessments by regulators. Equally important will be continued development of methods or approaches for consistently evaluating the residual uncertainty in any given read-across. Some residual level of uncertainty must be considered acceptable (see below), since even study data has a degree of associated uncertainty – be that due to species relevance, inherent experimental variability, or behavior of *in vitro* cell lines. So, although selected *in vitro* or indeed short term *in vivo* testing may be used to reduce the level of uncertainty, a residual level will persist.

To date, published approaches for systematically evaluating the uncertainty in a given read-across assessment have been limited (Patlewicz et al., 2015). In a qualitative framework for read-across uncertainty characterization (Blackburn and Stuard, 2014), the two major areas where uncertainty arises in read-across are outlined: first, the chemical/structural differences between the target and the source chemicals contributing to the dataset and second, the type, quality and consistency of the dataset itself. In each area, multiple features must be considered in determining the degree of confidence or level of residual uncertainty in a read-across. Structural differences between the target and source chemicals introduce uncertainty as a result of their impact on physical-chemical properties (which may result in differences in bioavailability) or reactivity and metabolism (which can lead to differences in toxicokinetics and potency). The potential for toxicokinetic differences between the target chemical as compared to the source chemical is a major area of uncertainty in SAR-based read-across and is of high concern to regulators charged with protecting public health. With regard to the quality and consistency of the dataset, in general the amount of residual uncertainty in the read-across is inversely proportional to the quantity, quality and continuity of the dataset contributed by source chemicals.

The uncertainty in these characteristics of a given read-across impacts the similarity justification for the source and target chemical as well as the completeness and conclusion of the read-across rationale (Schultz et al., 2015), and determining the level of uncertainty that is acceptable for a read-across prediction is still largely subjective and defined on a case-by-case basis. It is heavily influenced by the purpose of the read-across and the complexity of the endpoint being read across in addition to the severity of hazard exhibited in the dataset. For example, one could consider that a greater level of uncertainty associated with read-across for an endpoint such as skin irritation would be accepted versus an endpoint such as genotoxicity or developmental toxicity. While there is a general awareness and consensus across practitioners of SAR and regulators as to key features of an “ideal” or “low-to-no” uncertainty read-across, there is much less unanimity as to what constitutes “sufficient” as the information on these features becomes less robust or available. As a result, there is much less agreement on how the more limited data for that feature, which gives rise to more uncertainty, should be interpreted in the context of the read-across. This is because interpretation of the impact of a given feature of read-across, and its weight with respect to all the other features considered in the read-across, requires expert judgment. To complicate this interpretation, the impact of the level of robustness of data for any particular feature (i.e., the relative “weighting” across features) is somewhat dependent on the context of all the other information available for consideration in the read-across and therefore can change in different read-across situations or assessments. Therefore, continued work is needed to systematize and increase consistency and transparency of the expert judgment used to determine read-across uncertainty as well as to build consensus on the level of uncertainty that is acceptable for different applications of read-across.

## 5 The next steps – the future of read-across?

Having reviewed the current start of the art in terms of read-across, biological profiling and experiences to date with the regulatory acceptance of read-across, it is important to consider where this field should move in order to take advantage of this tool to the fullest extent.

### 5.1 Use of “fit for purpose” tools to support read-across

The tool box of read-across is continuously expanding. This includes databases, quality assurance criteria, tools to establish similarity, and programs for (quantitative) read-across. The use of proper cheminformatics is paramount to managing information and ensuring the predictivity of a read-across approach while minimizing uncertainty. More and more tools are becoming available for chemical structure-based read-across and hence it will be necessary to regularly update the inventory of “proper tools” for use based on expert consensus.

The examples brought together here, and in the accompanying paper (Zhu et al., 2016, this issue), show that the concept of biological similarity enhances read-across: If the target of interest and similar compounds with known effects have been tested in the same set of high-throughput assays, one can use a bio-fingerprint (i.e., a collective set of results from different assays) to profile the target against the tested compounds, and then compare the bio-fingerprint between the target and tested chemicals. One will have to prove the selected assays are relevant to the toxicological endpoint of interest, either from the understanding of the toxicological mechanism, e.g., as characterized by an Adverse Outcome Pathway (AOP), or from correlative data analysis, e.g., a significant relationship between the bio-fingerprint and the toxicological effect. If there is any data gap for generating the bio-fingerprint, i.e., lacking information for certain *in vitro* assays, one might use QSAR models to predict the results of the *in vitro* assays. When applying QSAR modeling, one should follow the respective OECD guidance for QSAR^23^ (ENV/JM/MONO(2004)24). For such an approach to have benefit, regulatory acceptance must also be forthcoming.

The increasing availability of biological databases will augment support of read-across and grouping by such data. The curation of these datasets and the respective data-sharing by companies, organizations and individual researchers needs to be further encouraged, possibly with some incentives. Alternatively, comprehensive profiling, typically by transcriptomics or metabolomics, of the biological effect of substances in complex systems representing many targets for perturbation can work in tandem, permitting support for similarity arguments within an individual assay.

The approaches presented here and in more detail in the accompanying paper (Zhu et al., 2016, this issue) cannot yet be considered routine approaches for read-across. However, they already promise, on a case-by-case basis, to support read-across arguments and should be considered when the respective test data are available or can be obtained with acceptable effort. For the future, more accessible standardized testing environments might offer bio-profiling of substances and thereby open the doors for enhanced read-across of substances that have not been broadly studied in the scientific literature.

### 5.2 Consistent approach to reporting and assessing read-across

With the majority of read-across failures under REACH resulting from a lack of supporting information or issues with scientific plausibility, it is important for the future of read-across that this is done in a structured and systematic way. One potential aid in encouraging practitioners of read-across to adopt this style of approach is the Read Across Assessment Framework (RAAF) developed by ECHA.

ECHA has recently published their RAAF, which describes the structured approach ECHA will use when evaluating the acceptability of read-across proposals included in REACH registrations (ECHA, 2015). The RAAF is meant to complement rather than replace the official REACH guidance documents on read-across for REACH registrations. It is acknowledged that read-across is a complex process that requires significant chemical and toxicological expertise and REACH registrants therefore are encouraged to use the RAAF as a tool to help them assess the quality of their read-across assessments prior to dossier submission. Noteworthy, the RAAF currently is limited to human health-related endpoints rather than ecotoxicity or environmental fate properties.

The RAAF includes an overall guidance document that, among other things, defines the six types of read-across scenarios that are recognized by ECHA and six appendices, one for each type of read-across scenario. The individual appendices provide the details of the critical elements that must be addressed in a robust read-across justification for that particular scenario. The six possible read-across scenarios identified by ECHA in the RAAF do not directly correlate with the 3 (main) ways that similarity between substances may be demonstrated (functional group, common precursors, and constant pattern in the changing of potency across the group) according to the REACH legal text and OECD guidance (OECD, 2014). Instead, the scenarios are characterized by 3 key features. The first feature is the number of source chemicals, i.e., either an analogue approach (data from one source chemical is read across to the target chemical) or a category approach (data from multiple source chemicals are read across to the target chemical) is used. The second feature is fundamental to the read-across hypothesis and requires the registrant to decide whether the toxic effect being investigated is caused by a common toxicant formed from both the target and source chemical or whether the same toxic effect is elicited by different toxicants between the target and source chemicals. Lastly, when multiple source chemicals are used, the read-across scenario must address whether there is a trend in the effect observed across source chemicals (e.g., a progression in potency across category members). By considering these 3 key features of a proposed read-across, one of six possible scenarios must be selected as the basis for the read-across hypothesis:

1. Analog approach / Common toxicant causing same effect
2. Analog approach / Same effect caused by different toxicants
3. Category approach / Common toxicant causing same effect/ effect varies (trend) across members
4. Category approach / Same effect caused by different toxicants / effect varies (trend) across members
5. Category approach / Common toxicant causing same effect/ effect does not vary across members
6. Category approach / Same effect caused by different toxicants/effect does not vary across members

ECHA has defined a set of “assessment elements” for each scenario that form the basis of their evaluation. These assessment elements refect the critical information/evidence that should be included in a scientifically robust justification for that type of read-across hypothesis (i.e., scenario). These critical scientific details are described and in many case examples of appropriate supportive evidence and illustrative examples are provided by ECHA. Also, importantly, often the concern or uncertainty that results in the read-across when that particular element is not addressed in the read-across justification is articulated. The level of detail available in the RAAF provides more transparency in terms of expectations and potential requirements for an acceptable read-across proposal than has been provided in other regulatory guidance documents to date. However, it is noteworthy that the expectations for supporting information are high.

While all of the scenarios have important hypothesis-specific considerations that must be addressed, there is some redundancy in the RAAF appendices since many of the critical scientific elements for read-across are relevant to all hypotheses or scenarios. Looking across the assessment elements listed in the RAAF for each of the read-across scenarios, they can be broadly sorted into four main categories:

– General elements applicable to any read-across hypothesis (scenarios 1-6)
– Element relevant only to the “Category approach” (scenarios 3-6)
– Specific elements applicable to the “Common toxicant hypothesis” (scenarios 1,3,5)
– Specific elements applicable to the “Different toxicant hypothesis” (scenarios 2,4,6)

General elements of a read-across justification that are relevant regardless of the read-across approach or hypothesis center around substance identity, linkage of chemical structure to the predicted property or effect, adequacy of data, and demonstrating lack of selection bias. Obviously, both the target and source substances have to be clearly identified and characterized with regard to potential impurity profiles and both structural similarities and any structural differences between these substances have to be addressed in the context of the effect (endpoint) being read across. Under the category approach, this includes making any relevant linkages between structural differences in the substances and any trends observed in the effect (end-point) being read across. In addition, the read-across justification should demonstrate that there is no bias in the read-across with respect to selection of source chemicals or selection of data included for the source chemicals (e.g., clearly define and provide search strategy and selection criteria for source chemicals). Lastly the source data must be adequate to meet the REACH information requirement data gap that is being filled using read-across. These requirements mirror well the assessment of past read-across case studies, where the above elements formed the major reasons for the rejection of the approach. If one is able to follow the RAAF when preparing a read-across justification, the future incidence of the issues faced to date should be reduced. However, the RAAF does also serve to highlight the complexity of read-across and may inadvertently lead to an increase in testing proposals where the barriers to read-across and the registration consequences are considered too high by a registrant.

Other critical scientific elements of the read-across justification, when using the Category approach, include clearly defining the inclusion/exclusion criteria for membership in the category and addressing consistency (and differences) of effect in the data matrix. The RAAF clearly establishes the expectation that for a category approach read-across, a data matrix should be included that is organized in a logical manner with respect to category members and their available data. The logical manner should include listing the source substances in a specific order based on a described (usually structural) parameter pivotal to category membership (e.g., in order of increasing alkyl chain length). All available relevant data for each category member should be included – not only for the endpoint being read across but also for related endpoints. Information on other endpoints (i.e., “anchor data”) can help solidify the read-across hypothesis by reducing uncertainty when the data are consistent or in the case of inconsistencies can help direct appropriate modification of the read-across hypothesis.

RAAF assessment elements applicable to the Common Toxicant or Different Toxicant hypotheses (regardless of whether analog or category approach) address the scientific aspects of how the source and target substances interact with the organism to impact the property being predicted by read-across. When the hypothesis is based on a “Common Toxicant”(e.g., common metabolite formed by both the sources and target chemical elicit the same effect), key scientific considerations include how the toxicant is formed, exposure of the biological target implicated in the effect being read across to the formed toxicant, and whether/how any other non-common substances formed that are different between the source and target chemicals impact the biological target/predicted property. When the hypothesis is based on different toxicants (i.e., no biotransformation – the parent source and target chemicals from the same chemical class are directly eliciting the effect being read across), key scientific considerations include addressing any other substances (e.g., different metabolites) and their potential to influence the predicted property, providing a rationale for how the different toxicants are acting through a common underlying mechanism to elicit the effect being read across, examining the quantitative aspects of this common underlying mechanism with regard to any differences in exposure of the biological target based on ADME/TK, and evaluating the relevance of any other effects observed in the data (not necessarily linked to the hypothesis) on the predicted property.

As described here, ECHA's published RAAF contains a wealth of useful information that could serve as a starting point for development of a more consistent and transparent approach for constructing scientifically robust read-across assessments. While it remains to be seen whether the specific level of detail and supporting evidence for some of the critical elements as defined by ECHA can be realistically achieved, the RAAF nonetheless provides an articulated “best practices” target for the execution and documentation of structure-activity relationship based read-across. It should be recognized that the RAAF does not completely cover the use of read-across as it does not specifically address the use of read-across for environmental fate and ecotoxicity endpoints. While it also does not adequately address UVCB substances, several of the elements in the RAAF can be generally applied to UVCB substances, but some (including those pertaining to substance identity) are more difficult to apply. In addition, while the use of novel data such as omics and bio-profiling are encouraged, the reporting and the understanding of the assessment of this data is not implicit in the RAAF. These therefore represent future opportunities to provide an input into how best practices can be established.

The RAAF currently requires structural similarity of chemicals used for read-across purposes. Although this may be a prudent starting point, from a biological point of view this is not necessarily a prerequisite to perform a valid read-across. If chemically dissimilar structures would activate a particular AOP, and this can be convincingly demonstrated, then this could provide a basis for read-across consideration. If further data, e.g., from omics technologies, would demonstrate a clearly similar overall profile of biological activity, then read-across could be justified, even if the chemical structures of the compounds under consideration are not completely identical. Examples for such an approach using *in vivo* metabolomics are provided in van Ravenzwaay et al. (2012).

## 6 Conclusions

This paper has attempted to lay out the current state of the art of read-across and, based on the analysis of its effectiveness under the EU REACH Regulation, highlights the areas that now should become a priority for further work to ensure that read-across continues to be an effective way to characterize the hazards of substances without triggering the need for extensive testing programs. Several areas now appear to be clear focal points for more work:

– Finding an effective way to make use of the ECHA RAAF to guide the creation of categories/analog groupings and support read-across within these: The RAAF is an extensive modular document detailing the critical scientific aspects associated with different types of read-across hypotheses. Developing a companion tool with a reporting function that could serve as a category/analogue approach justification document would be of significant utility to a broad range of read-across practitioners. This tool may also assist in a better assessment of uncertainty within the use of read-across and allow practitioners to be more proactive in identifying ways to address uncertainty (e.g., through risk assessment or triggering additional data generation).
– Continued analysis and reporting of case studies, including those that have been successfully accepted by regulatory authorities: Availability of positive examples of robust read-across case studies can serve as effective models to help drive consistency in read-across assessments developed by industry. We must continue to learn from the experiences of others.
– Identify the best practices for using biological profiling/bio-informatics tools to support establishing similarity of source and target chemicals in read-across and, potentially, for predicting endpoints in their own right: There is a lot of movement in the area of biological profiling, and ensuring that these techniques are used in a robust manner that is reliable and transparent is critical. Failure to derive some best practices for their use will likely lead to significant uncertainty on how to use these tools effectively and also create some mistrust in the data where they are not used appropriately.

## Supplementary Material

Supplementl file

## Figures and Tables

**Tab. 1:** Reasons for the rejection of the use of read-across in disseminated compliance check decisions published on the ECHA website by July 31, 2015

Reason for rejection	No. of cases
Unclear substance identity, not possible to ascertain structural similarity – A significant issue for UVCB substances with a severe impact on large UVCB categories using a combination of read-across and targeted testing	48
Lack of sufficient evidence to substantiate assumptions made within read-across justifications – Including lack of data on analogues provided in dossier	43
Read-across to inappropriate data – For example read-across to a reproductive screening study to address higher tier reproductive and developmental study requirements	5
Lack of scientific plausibility – Disagreement with hypothesis, data not supportive of arguments presented, too much uncertainty – This often combined with the lack of sufficient evidence/information	20

**Tab. 2:** Case study summaries

Case no.	Substance(s)	Decision no.
Case 1	2-diethylaminoethanol	CCH-D-2114289315-43-01/F
Case 2	reaction mass of Amides, rape-oil, N-(hydroxyethyl), ethoxylated and Glycerol, ethoxylated	CCH-D-0000005614-74-01/F
Case 3	dipropylene glycol methyl ether acetate	CCH-D-0000001716-72-04/F
Case 4	cycloexyldimethoxymethylsilane	TPE-D-0000003049-75-05/F
Case 5	ethylene carbonate	CCH-D-2114290256-46-01/F
Case 6	4-hydroxy-4-methylpentan-2-one	CCH-D-2114288084-45-01/F
Case 7	2-butene	CCH-D-0000004339-69-03/F
Case 8	dibutyl fumarate	CCH-D-2114292038-46-01/F
Case 9	hydrogenated dimerization products of 1-decene, 1-dodecene and 1-octene	CCH-D-0000002118-79-10/F
Case 10	cobalt compounds	TPE-D-0000003367-71-04/F
Case 11	higher alpha olefins	TPE-D-0000003868-59-04/F

## Footnotes

1 http://www.oecd.org/chemicalsafety/risk-assessment/theoecdqsartoolbox.htm

2 http://www.echemportal.org/echemportal/index?pageID=0&request_locale=en

3 http://bit.ly/1RdJ70G

4 http://echa.europa.eu/information-on-chemicals/registered-substances

5 https://www.ebi.ac.uk/chembl/

6 https://ncats.nih.gov/tox21

7 http://www.epa.gov/chemical-research/toxicity-forecasting

8 http://echa.europa.eu/

9 http://ec.europa.eu/environment/chemicals/reach/legislation_en.htm

10 emphasis added

11 https://echa.europa.eu/documents/10162/13643/substance_id_en.pdf

12 https://echa.europa.eu/documents/10162/13632/information_requirements_r6_en.pdf

13 https://eurl-ecvam.jrc.ec.europa.eu/databases/jrc-qsar-model-database-and-qsar-model-reporting-formats

14 http://www.oecd.org/env/ehs/oecdquantitativestructure-activityrelationshipsprojectqsars.htm

15 http://www.antares-life.eu

16 http://www.antares-life.eu/fles/ANTARES_final_report.pdf

17 http://www.oecd.org/env/ehs/risk-assessment/theoecdqsartoolbox.htm

18 http://actor.epa.gov/actor/toxcastdbata.jsp

19 http://www.lincsproject.org

20 https://www.nc3rs.org.uk/arrive-guidelines

21 www.ebtox.com

22 https://ntp.niehs.nih.gov/pubhealth/hat/noms/index-2.html

23 http://www.oecd.org/officialdocuments/publicdisplaydocumentpdf/?cote=env/jm/mono(2004)24&doclanguage=en
