# Supplementary material for: t4 report: Toward Good Read-Across Practice (GRAP) Guidance
Source: ALTEX. Author manuscript; Available in PMC 2017 Sep 1. (PMC5581000; doi:10.14573/altex.1601251)
Supplement: Supplementl file [file NIHMS834020-supplement-Supplementl_file.pdf]

Ball et al.:

t⁴ report

Toward Good Read-Across Practice (GRAP) Guidance

Supplementary Data

Decision number	Substance name	EC #	CAS #	Ac-cepted	Sub-stance ID	Lack of suf-ficient infor-mation	Inap-pro-priate data	Sci-entific plausi-bility
CCH-D-2114290256-46-01/F	Ethylene carbonate	202-510-0	96-49-1			x		x
CCH-D-2114288615-38-01/F	Magnesium, bis(2-hydro-xybenzoato-o1,o2)-, ar, ar'-di-c14-18alkyl derivs.	931-371-5	171171-80-5			x	x	
CCH-D-2114288112-54-01/F	Benzoic acid, 2-hydroxy-, mono-c14-18-alkyl derivs., calcium salts (2:1)	931-276-9	114959-46-5	x				
CCH-D-2114288751-40-01/F	Dibutyl maleate	203-328-4	105-76-0			x		
CCH-D-2114289315-43-01/F	2-Diethylaminoethanol	202-845-2	100-37-8			x		
CCH-D-2114288084-45-01/F	4-Hydroxy-4-methyl-pentan-2-one	204-626-7	123-42-2					x
CCH-D-0000005614-74-01/F	Reaction mass of amides, rape-oil, N-(hydroxyethyl), ethoxylated and glycerol, ethoxylated	932-164-2				x		
CCH-D-2114289309-36-01/F	Hexahydro-4-methylphthalic anhydride	243-072-0	19438-60-9			x		x
CCH-D-0000004339-69-03/F	2-Butene	203-452-9	107-01-7					x
CCH-D-2114288054-48-01/F	Butan-2-ol	201-158-5	78-92-2			x		x
CCH-D-0000005483-73-04/F	Tert-butyl acetate	208-760-7	540-88-5					x
CCH-D-0000004026-82-05/F	Phosphorodithioic acid, mixed O,O-bis(iso-bu and pentyl) esters, zinc salts	270-608-0	68457-79-4	x*				
CCH-D-0000004032-89-05/F	Zinc bis[O,O-bis(2-ethyl-hexyl)] bis(dithiophosphate)	224-235-5	4259-15-8	x*				
CCH-D-0000005192-80-02/F	Benzotriazole	202-394-1	95-14-7			x		
CCH-D-0000004930-75-04/F	Prop-2-yn-1-ol	203-471-2	107-19-7					x
CCH-D-0000004884-64-04/F	Ethylenediamine, ethoxy-lated and propoxylated	500-047-1	26316-40-5			x		

* Decision withdrawn by ECHA; (x) Partial acceptance of read-across

This is an Open Access article distributed under the terms of the Creative Commons Attribution 4.0 International license (http://creativecommons.org/licenses/by/4.0/), which permits unrestricted use, distribution and reproduction in any medium, provided the original work is appropriately cited.

http://dx.doi.org/10.14573/altex.1601251s

Decision number	Substance name	EC #	CAS #	Accepted	Substance ID	Lack of sufficient information	Inappropriate data	Scientific plausibility
CCH-D-0000004262-82-05/F	Propylene carbonate	203-572-1	108-32-7			x		
CCH-D-0000003802-77-04/F	Isophthaloyl dichloride	202-774-7	99-63-8			x		
CCH-D-0000003782-71-03/F	Bismuth vanadium tetraoxide	237-898-0	14059-33-7			x		
CCH-D-0000002563-76-10/F	2,4-Di-tert-butylphenol	202-532-0	96-76-4					
CCH-D-0000003713-76-05/F	4-Hydroxy-2,2,6,6-tetramethylpiperidine-1-ethanol	258-132-1	52722-86-8			x	x	
CCH-D-0000002210-93-09/F	Geraniol	203-377-1	106-24-1			x	x	
CCH-D-0000003824-71-06/F	Alkenes, C7-9, hydroformylation products, distn. residues, heavy cracked fraction	308-482-7	98072-31-2					x
CCH-D-0000003878-58-03/F	2-Propenoic acid, butyl ester, reaction products with butadiene, sulfur and tri-Ph phosphite	300-339-7	93925-37-2			x		
CCH-D-0000001733-76-10/F	Bis(nonylphenyl)amine	253-249-4	36878-20-3				x	
CCH-D-0000002643-76-02/F	67828-72-2_Master_strontium 4-[(4-chloro-5-methyl-2-sulphonatophenyl) azo]-3-hydroxy-2-naphthoate (1:1),	267-291-6	67828-72-2					x
CCH-D-0000003992-66-02/F	(3-Chloropropyl)triethoxysilane	225-805-6	5089-70-3				x	
CCH-D-0000002963-68-04/F	Zirconium praseodymium yellow zircon	269-075-7	68187-15-5			x		
CCH-D-0000003135-82-03/F	Hydrocarbons, C4, 1,3-butadiene- and isobutene-free	306-004-1	95465-89-7			x		
CCH-D-0000003136-80-03/F	Hydrocarbons, C4, steam-cracker distillate	295-405-4	92045-23-3			x		
CCH-D-0000003450-84-02/F	2-methylbutane	201-142-8	78-78-4			x		
CCH-D-0000003196-74-05/F	Dibutyltin oxide	212-449-1	818-08-6			x		
CCH-D-0000003124-85-04/F	Fatty acids, hydrogenated tallow, distn. residues	274-307-5	70084-85-4			x		
CCH-D-0000003129-75-04/F	Reaction mass of ethyl benzene and xylene	905-588-0				x		
CCH-D-0000003128-77-04/F	Reaction mass of 2-methylbutyl acetate and pentyl acetate	908-918-1				x		
CCH-D-0000003111-92-04/F	Reaction mass of ethylbenzene and m-xylene and p-xylene	905-562-9				x		
CCH-D-0000003117-80-04/F	Hydrocarbons, C5, n-alkanes, isoalkanes	921-577-3				x		
CCH-D-0000003116-82-04/F	Hydrocarbons, C6, n-alkanes, iso-alkanes, cyclics, n-hexane rich	925-292-5				x		
CCH-D-0000001978-58-08/F	2-octyldodecan-1-ol	226-242-9	5333-42-6			x		

Decision number	Substance name	EC #	CAS #	Ac-cepted	Sub-stance ID	Lack of suf-ficient infor-mation	Inap-pro-priate data	Sci-entific plausi-bility
CCH-D-0000002025-86-08/F	Sodium ethylenesulphonate	221-242-5	3039-83-6		x			
CCH-D-0000002577-67-04/F	Cyclohexanone	203-631-1	108-94-1			x		
CCH-D-0000002614-77-03/F	Tall oil	232-304-6	8002-26-4		x	x		
CCH-D-0000002118-79-10/F	Hydrogenated dimerization products of 1-decene, 1-dodecene and 1-octene	700-308-1				x		x
CCH-D-0000001990-72-06/F	2-Dimethylaminoethyl methacrylate	220-688-8	2867-47-2			x		x
CCH-D-0000002304-84-04/F	Vinyl 2-ethylhexanoate	202-297-4	94-04-2			x		x
CCH-D-0000002254-81-04/F	Substance name and link to registration dossier not given as, due to exceptional circumstances specific to a registrant's situation, it has been established that such link would cause harm to commercial interests.				x	x		
CCH-D-0000002397-69-02/F	Dichloro(dimethyl)silane	200-901-0	75-78-5	(x)				x
CCH-D-0000002289-68-04/F	1,1,1,3,3,3-Hexamethyl-disilazane	213-668-5	999-97-3					x
CCH-D-0000002301-90-03/F	Vinyl neononanoate	259-160-7	54423-67-5					x
CCH-D-0000001699-60-07/F	Allyl alcohol	203-470-7	107-18-6			x		x
CCH-D-0000002181-86-02/F	00000 RP002- Fe(III)HBED	700-327-5	1061328-86-6			x		
CCH-D-0000001422-85-05/F	Camphene	201-234-8	79-92-5			x		x
CCH-D-0000001796-64-04/F	Ethanol, 2-methoxy-, manu-facture of, by-products from, esters with boric acid	310-290-3	161907-80-8		x			
CCH-D-0000001777-64-03/F	Rosin, fumarated, reaction products with glycerol and pentaerythritol	296-047-1	92202-14-7		x			
CCH-D-0000001776-66-03/F	Rosin, fumarated, reaction products with formaldehyde	305-795-0	95009-65-7		x			
CCH-D-0000001774-70-03/F	Reaction mass of resin acids and rosin acids, hydro-genated, sodium salts and sodium [1R-(1a,4aß,10aa)]-1,2,3,4,4a,9,10,10a-octahydro-7-isopropyl-1,4a-dimethylphen-anthren-1-carboxylate	915-568-3			x			
CCH-D-0000001773-72-03/F	Resin 835A	911-238-8			x			
CCH-D-0000001772-74-03/F	Resin acids and rosin acids, hydrogenated, esters with glycerol	266-042-9	65997-13-9		x			
CCH-D-0000001771-76-03/F	Rosin, maleated	232-480-4	8050-28-0		x			
CCH-D-0000001770-78-03/F	Resin acids and rosin acids, hydrogenated, esters with pentaerythritol	264-848-5	64365-17-9		x			

Decision number	Substance name	EC #	CAS #	Ac-cepted	Sub-stance ID	Lack of suf-ficient infor-mation	Inap-pro-priate data	Sci-entific plausi-bility
CCH-D-0000001765-69-03/F	Rosin, hydrogenated	266-041-3	65997-06-0		x			
CCH-D-0000001764-71-03/F	Reaction mass of resin acids and rosin acids, hydrogenated, potassium salts and potassium [1R-(1a,4aß,10aa)]-1,2,3,4,4a,10,10a, octahydro-7-isopropyl-1, 4a dimethylphenanthren-1-carboxylate	915-657-7			x			
CCH-D-0000001759-62-03/F	R-F-P 94581-15-4, resin acids and Rosin acids, fumarated, esters with pentaerythritol	305-514-1	94581-15-4		x			
CCH-D-0000001775-68-03/F	Fatty acids, tall-oil, oligomeric reaction products with maleic anhydride and rosin, calcium magnesium zinc salts	500-451-8	160901-14-4		x			
CCH-D-0000001769-61-03/F	Resin acids and rosin acids, esters with glycerol	232-482-5	8050-31-5		x			
CCH-D-0000001767-65-03/F	Resin acids and rosin acids, esters with triethylene glycol	232-478-3	8050-25-7		x			
CCH-D-0000001763-73-03/F	Resin acids and rosin acids, calcium zinc salts	269-825-3	68334-35-0		x			
CCH-D-0000001762-75-03/F	Resin acids and rosin acids, sodium salts	263-144-5	61790-51-0		x			
CCH-D-0000001761-77-03/F	Rosin, reaction products with formaldehyde	293-659-0	91081-53-7		x			
CCH-D-0000001758-64-03/F	Resin acids and rosin acids, potassium salts	263-142-4	61790-50-9		x			
CCH-D-0000001757-66-03/F	Magnesium (1R,4aR,4bR,10aR)-1, 4a-dimethyl-7-propan-2-yl-2,3,4,4b,5,6,10,10a-octahydrophenanthrene-1-carboxylate	270-461-2	68440-56-2		x			
CCH-D-0000001756-68-03/F	Calcium (1R,4aR,4bR,10aR)-1,4a-dimethyl-7-propan-2-yl-2,3,4,4b,5,6,10,10a-octahydrophenanthrene-1-carboxylate	232-694-8	9007-13-0		x			
CCH-D-0000001716-72-04/F	Dipropylene glycol methyl ether acetate	406-880-6	88917-22-0			x		x
CCH-D-0000001941-75-02/F	Disodium metasilicate	229-912-9	6834-92-0			x		
CCH-D-0000001702-81-02/F	Decane	204-686-4	124-18-5		x			
CCH-D-0000001655-72-03/F	Dodecane	203-967-9	112-40-3		x			
CCH-D-0000001649-65-03/F	Undecane	214-300-6	1120-21-4		x			

Decision number	Substance name	EC #	CAS #	Ac-cepted	Sub-stance ID	Lack of suf-ficient infor-mation	Inap-pro-priate data	Sci-entific plausi-bility
CCH-D-0000001643-77-03/F	Hydrocarbons, C13-C18, n-alkanes, isoalkanes, cyclics, <2% aromatics	928-253-0			x			
CCH-D-0000001641-81-03/F	Hydrocarbons, C10-C14, n-alkanes, isoalkanes, <2% aromatics	920-274-3			x			
CCH-D-0000001639-66-03/F	Hydrocarbons, C10-C13, n-alkanes, <2% aromatics	929-018-5			x			
CCH-D-0000001638-68-03/F	Isododecane	297-629-8	93685-81-5		x			
CCH-D-0000001633-78-03/F	Tetradecane	211-096-0	629-59-4		x			
CCH-D-0000001631-82-03/F	Hydrocarbons, C14-C20, n-alkanes, aromatics (<2%)	923-583-1			x			
CCH-D-0000001629-67-03/F	Hydrocarbons, C16-C20, n-alkanes, isoalkanes, cyclics, aromatics (<2%)	919-029-3			x			
CCH-D-0000001628-69-03/F	Hydrocarbons, C13-C15, n-alkanes, isoalkanes, cyclics, <2% aromatics	917-488-4			x			
CCH-D-0000001624-77-03/F	Isohexadecane	297-628-2	93685-80-4		x			
CCH-D-0000001623-79-03/F	Hydrocarbons, C14-C20, n-alkanes, isoalkanes, <2% aromatics	931-265-9			x			
CCH-D-0000001618-70-03/F	Hydrocarbons, C14-C18, n-alkanes, isoalkanes, cyclics, <2% aromatics	927-632-8			x			
CCH-D-0000001640-83-03/F	Substance name and link to registration dossier not given as, due to exceptional circumstances specific to a registrant's situation, it has been established that such link would cause harm to commercial interests.				x			
CCH-D-0000001635-74-03/F	Hydrocarbons, C10-C13, n-alkanes, isoalkanes, cyclics, < 2% aromatics	918-481-9			x			
CCH-D-0000001626-73-03/F	920-134-1	920-134-1			x			
CCH-D-0000001650-82-03/F	Hydrocarbons, C11-C14, n-alkanes, <2% aromatics	924-803-9			x			
CCH-D-0000001644-75-03/F	Tridecane	211-093-4	629-50-5		x			
CCH-D-0000001637-70-03/F	Hydrocarbons, C10-C13, n-alkanes, isoalkanes, cyclics, aromatics (2-25%)	919-164-8						
CCH-D-0000001627-71-03/F	Hydrocarbons, C14-C18, n-alkanes, isoalkanes, cyclics, aromatics (2-30%)	920-360-0			x			
CCH-D-0000001625-75-03/F	Hydrocarbons, C14-C19, isoalkanes, cyclics, <2% aromatics	920-114-2			x			

Decision number	Substance name	EC #	CAS #	Ac-cepted	Sub-stance ID	Lack of suf-ficient infor-mation	Inap-pro-priate data	Sci-entific plausi-bility
CCH-D-0000001622-81-03/F	Hydrocarbons, C11-C14, isoalkanes, cyclics, <2% aromatics	927-285-2			x			
CCH-D-0000001617-72-03/F	Hydrocarbons, C14-C17, n-alkanes, <2% aromatics	917-828-1			x			
CCH-D-0000001604-79-03/F	Hydrocarbons, C9-C12, n-alkanes, isoalkanes, cyclics, aromatics (2-25%)	919-446-0			x			
CCH-D-0000001329-73-04/F	Substance name and link to registration dossier not given as, due to exceptional circumstances specific to a registrant's situation, it has been established that such link would cause harm to commercial interests.					x		
CCH-D-0000001260-89-04/F	S190700	443-510-2				x		
CCH-D-0000001212-90-05/F	Vegeflux soy	483-980-6				x		
CCH-D-0000001202-91-03/F	Substance name not yet available, as the public name to be used in the registered substances web-page is being determined.			(x)				
CCH-D-2114289967-22-01/F	Buta-1,2-diene	209-674-2	590-19-2					x
CCH-D-2114289108-42-01/F	Dimethylamine	204-697-4	124-40-3			x		
CCH-D-2114292038-46-01/F	Dibutyl fumarate	203-327-9	105-75-9			x		x
			**Total Count**	**5**	**48**	**43**		**20**

* Decision withdrawn by ECHA; (x) Partial acceptance of read-across
